# RNA Interference-Mediated Silencing of *HbREF* and *HbSRPP* Genes Reduces Allergenic Protein Content While Maintaining Rubber Production in *Hevea brasiliensis*

**DOI:** 10.3390/ijms26209944

**Published:** 2025-10-13

**Authors:** Thanyarat Kuasuwan, Methaporn Meethong, Napassawan Inaek, Panumas Puechpon, Sumalee Obchoei, Phanthipha Runsaeng

**Affiliations:** 1 Division of Health and Applied Sciences, Faculty of Science, Prince of Songkla University, Hat Yai 90110, Songkhla, Thailandnapassawan95243@gmail.com (N.I.); spectrum.ppp@gmail.com (P.P.);; 2 Center of Excellence for Biochemistry, Faculty of Science, Prince of Songkla University, Hat Yai 90110, Songkhla, Thailand

**Keywords:** RNA interference, allergenic proteins, para rubber, latex

## Abstract

Allergenic proteins in natural rubber latex (NRL) pose significant health risks, particularly in rubber gloves. This study evaluated RNA interference (RNAi) technology for silencing *HbREF* (rubber elongation factor) and *HbSRPP* (small rubber particle protein) genes in *Hevea brasiliensis* to reduce latex allergen content. Double-stranded RNA (dsRNA) targeting these genes demonstrated high stability at 25–37 °C for 6 h and under UV/outdoor conditions for 72 h, but degraded rapidly above 50 °C. Among the three delivery methods tested, direct injection achieved the highest efficiency (>90% gene silencing within 12 h), followed by root drenching (54–84%) and foliar spray (46–70%). *HbREF* silencing achieved 98–99% expression reduction within 3 h, while *HbSRPP* showed dose-dependent responses (70–90% silencing) without off-target effects. Gene silencing affected downstream rubber synthesis genes *HbCPT* (cis-prenyltransferase) and *HbRME* (rubber membrane elongation protein) (37–58% reduction) while upstream genes remained unaffected. *HbREF* silencing reduced Hev b1 allergen by 64.04% and Hev b3 by 12.51%, whereas *HbSRPP* silencing decreased Hev b3 by 71.54% and Hev b1 by 13.48%. Both treatments caused only a 11–13% reduction in dry rubber content. This RNAi approach effectively reduces major latex allergens while maintaining rubber production, demonstrating commercial potential for developing hypoallergenic rubber products through precision agriculture biotechnology.

## 1. Introduction

The para rubber tree (*Hevea brasiliensis*) represents a critical economic resource globally, with natural rubber latex serving as an indispensable raw material for numerous medical and industrial applications. Natural rubber products remain in high demand globally, particularly in the medical sector where latex-based materials such as gloves and protective equipment are indispensable [1,2,3]. Thailand, as one of the world’s largest producers, plays a central role in meeting this demand.

Despite its commercial importance, natural rubber latex presents significant health challenges due to the presence of allergenic proteins that can trigger severe immune responses in sensitive individuals. Natural rubber latex, a colloidal dispersion of cis-1,4-polyisoprene particles suspended in an aqueous serum, contains approximately 1–2% proteins by weight, of which more than 15 have been identified as potential allergens [2,4,5]. These latex protein allergies manifest across a spectrum of clinical presentations, ranging from mild contact dermatitis and localized skin irritation to severe systemic anaphylaxis that can be life-threatening [6,7]. The prevalence of latex allergies among healthcare workers and patients with frequent medical device exposure has reached concerning levels, necessitating urgent solutions to mitigate this occupational and medical hazard.

Current approaches to reduce allergenic protein content in natural rubber involve post-harvest processing methods, including physical separation techniques such as centrifugation, chemical treatments using surfactants and leaching agents, and enzymatic degradation using proteases [8,9]. However, these conventional methods suffer from significant limitations, including incomplete allergen removal, substantial processing costs, time-intensive procedures, and considerable losses of rubber particles during treatment. Furthermore, these post-harvest interventions often compromise the physical and mechanical properties of the final rubber products, limiting their effectiveness as comprehensive solutions.

The identification and characterization of major latex allergens have revealed that rubber elongation factor (REF, also designated Hev b1) and small rubber particle protein (SRPP, designated Hev b3) constitute the most clinically significant allergenic proteins in natural rubber latex [10,11]. These proteins are integral components of the rubber biosynthesis machinery, with REF playing a crucial role in rubber particle elongation and SRPP contributing to the stability and formation of small rubber particles. Their essential functions in rubber production and their location within rubber particles make them particularly challenging targets for conventional removal methods, as their elimination through processing would inevitably compromise rubber yield and quality. Although other allergen-associated proteins exist in *Hevea*, *HbREF* and *HbSRPP* were specifically selected as targets in this study due to their status as the major latex allergens (Hev b1 and Hev b3) and their central roles in rubber particle formation. Focusing on these two proteins allows for a detailed investigation of their specific contributions to both rubber biosynthesis and allergenic protein synthesis, providing critical insights that can guide future studies on additional latex allergens.

Recent advances in plant molecular biology have opened new avenues for addressing this challenge through targeted genetic approaches. RNA interference (RNAi) technology has emerged as a powerful tool for post-transcriptional gene silencing, offering the potential to reduce specific protein expression without permanent genetic modifications. Preliminary investigations using RNAi to target *SRPP* in alternative rubber-producing species have provided valuable insights into the feasibility of this approach. Studies in dandelion (*Taraxacum kok-saghyz*) demonstrated that *SRPP* silencing resulted in reduced rubber yield and altered molecular mass distribution, indicating the protein’s critical role in rubber biosynthesis [12]. Similarly, research in *Taraxacum brevicorniculatum* revealed that RNAi-mediated *SRPP* knockdown decreased rubber production without affecting average molecular mass, while rubber particle morphology remained largely unchanged [13].

Investigations targeting *REF* have yielded complementary findings, with RNAi-mediated silencing in *T. brevicorniculatum* resulting in the reduced molecular mass of cis-1,4-polyisoprene without affecting overall rubber particle stability [14]. These studies collectively suggest that while REF and SRPP are essential for optimal rubber production, their partial reduction through RNAi may be tolerable and could potentially provide a pathway to reduce allergenic protein content while maintaining acceptable rubber yields.

Despite these promising preliminary findings, comprehensive studies investigating RNAi-mediated silencing of allergenic proteins in commercial rubber trees remain limited. Critical questions regarding the stability of double-stranded RNA (dsRNA) under field conditions, optimal delivery methods for effective gene silencing, and the quantitative relationship between gene silencing efficiency and allergenic protein reduction requires systematic investigation. Furthermore, the downstream effects of *REF* and *SRPP* silencing on other components of the rubber biosynthesis pathway and overall latex composition need thorough characterization to ensure that interventions do not inadvertently compromise rubber quality or tree health.

The present study addresses these knowledge gaps by conducting a comprehensive evaluation of RNAi-mediated silencing of *HbREF* and *HbSRPP* genes in *H. brasiliensis*. Our objectives include the following: (1) assessing the environmental stability of dsRNA under conditions relevant to field applications, (2) comparing the effectiveness of different dsRNA delivery methods, (3) quantifying the impact of gene silencing on target and non-target gene expression, (4) measuring the effects on dry rubber content (DRC), and (5) determining the extent of allergenic protein reduction achievable through this approach. The insights gained from this research will provide essential foundations for developing biotechnological strategies to produce hypoallergenic natural rubber while maintaining commercial viability, potentially revolutionizing the safety profile of latex-based medical and industrial products.

## 2. Results

### 2.1. dsRNA Stability Under Different Conditions

**Figure 1:**
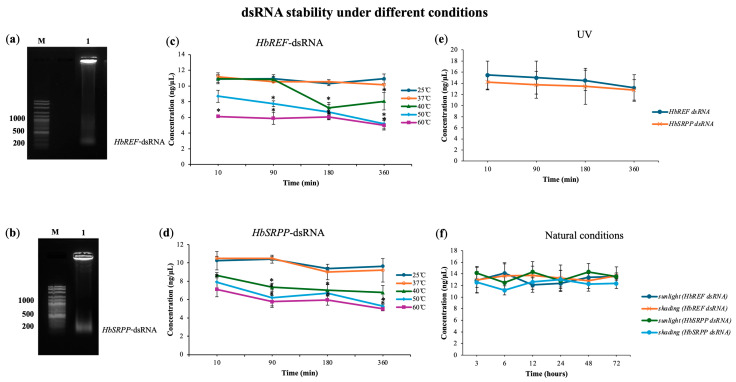
Stability analysis of dsRNA targeting *HbREF* and *HbSRPP* genes under various environmental conditions. The migration of (**a**) *HbREF*-specific dsRNA and (**b**) *HbSRPP*-specific dsRNA in agarose gel electrophoresis was compared against a DNA marker to estimate fragment size. Temperature stability profiles showing dsRNA concentration for (**c**) *HbREF* and (**d**) *HbSRPP* following incubation at 25–60 °C in a water bath at different time intervals. (**e**) UV stability of *HbREF* and *HbSRPP* dsRNA under continuous UV irradiation at various exposure times. (**f**) Environmental stability of dsRNA under natural sunlight and shade conditions. dsRNA concentrations were quantified using a SPECTROstar Nano microplate reader with LVis Plate technology. Data represent mean ± standard error from three independent experiments. Statistical significance was determined by one-way ANOVA (* *p* < 0.05).

The stability of dsRNA in various environmental conditions was assessed, given its potential application as a foliar spray or root drenching treatment. Tests were conducted under room-temperature and high-temperature conditions ranging from 25 °C to 60 °C. The results showed that dsRNA remains highly stable between 25 °C and 37 °C for up to 360 min. However, at temperatures of 40 °C, rapid degradation began after 180 min (27.83% for *HbREF*-dsRNA and 33.21% for *HbSRPP*-dsRNA) whereas at temperatures of 50 °C, rapid degradation began after 180 min (33%), with more than 45% of the dsRNA degrading within 6 h (Figure 1). Additionally, at temperatures of 60 °C, rapid degradation began after 10 min, with more than 50% of the dsRNA degrading within 360 min. Additionally, dsRNA was found to be relatively stable under 6 h of continuous ultraviolet (UV) irradiation, indicating its resilience in typical environmental conditions involving high temperatures and sunlight exposure. In outdoor conditions, regardless of exposure to sunlight or shade, dsRNA did not degrade within 72 h. Therefore, dsRNA can be reliably used in experiments involving direct injection, foliar spray, or root drenching formats.

### 2.2. Comparison of Delivery Techniques

**Figure 2:**
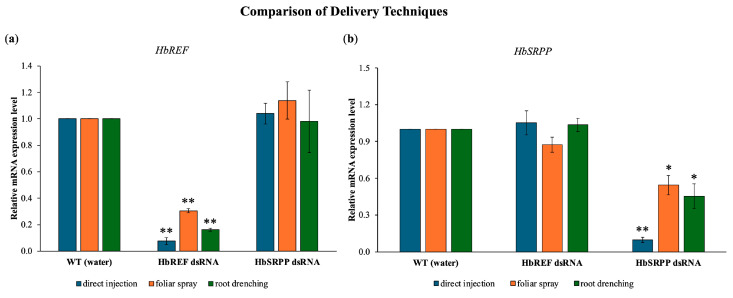
Comparative analysis of dsRNA delivery systems for RNAi-mediated gene silencing in *H. brasiliensis*. Quantitative RT-PCR analysis showing relative mRNA expression levels of (**a**) *HbREF* and (**b**) *HbSRPP* genes post-treatment with gene-specific dsRNA delivered through direct stem injection, foliar spray application, or root drenching methods. Expression levels were normalized to housekeeping genes and calculated using the 2^−ΔΔCt^ method relative to mock-treated controls. Data represent mean ± standard error from three independent biological replicates. Statistical analysis performed using one-way ANOVA with Tukey’s multiple comparison test (* *p* < 0.05, ** *p* < 0.01).

The study introduced dsRNA into para rubber trees using three methods including direct injection, foliar spray, and root drenching. It was found that all three methods were capable of delivering dsRNA into the cells, leading to the knockdown of the *HbREF* and *HbSRPP* genes. However, the foliar spray method only achieved gene expression inhibition of 69.5% and 45.6%, respectively, with clear gene silencing observed starting from 12 h post-application (Figure 2). The root drenching method also successfully delivered dsRNA into the cells, as evidenced by a significant reduction in the expression levels of *HbREF* and *HbSRPP* in the leaves, reaching reductions of 83.7% and 54.6%, respectively, after 24 h. The most effective method for delivering dsRNA and achieving gene silencing was direct injection, which resulted in a 92.4% reduction in *HbREF* expression and a 90.4% reduction in *HbSRPP* expression.

### 2.3. Expression of HbREF and HbSRPP Genes After RNAi Induction

**Figure 3:**
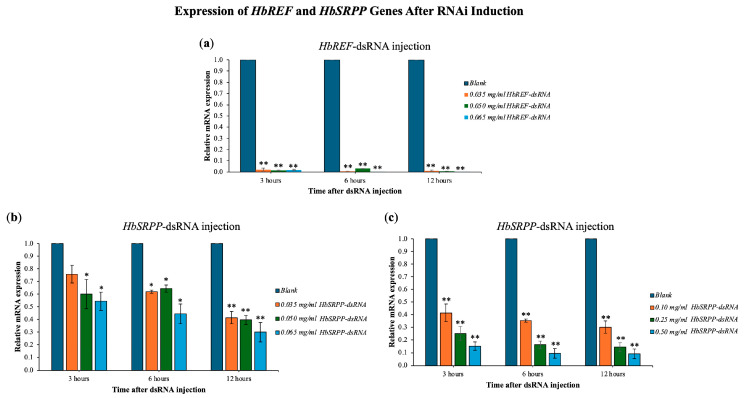
Dose-dependent gene silencing efficiency. Quantitative RT-PCR analysis of relative mRNA expression levels following treatment with gene-specific dsRNA at various concentrations. (**a**) *HbREF* mRNA expression after dsRNA treatment; (**b**,**c**) *HbSRPP* mRNA expression following dsRNA treatment. Gene expression was normalized to housekeeping genes and calculated relative to untreated control plants using the 2^−ΔΔCt^ method. Data represent mean ± standard error from three independent biological replicates. Statistical significance was determined by one-way ANOVA with post hoc analysis (* *p* < 0.05, ** *p* < 0.01).

The study examined the expression of *HbREF* and *HbSRPP* genes in para rubber tree leaves following RNAi induction with various concentrations of dsRNA. Results showed that dsRNA targeting *HbREF* reduced its gene expression by 98–99%, with a reduction observed as early as 3 h post-injection (Figure 3). For the *HbSRPP* gene, the highest dsRNA concentration of 0.065 mg/mL reduced gene expression by up to 70%, with the most significant decrease occurring at 12 h post-injection. Subsequent experiments with higher *HbSRPP*-dsRNA concentrations (0.10, 0.25, and 0.50 mg/mL) demonstrated further reductions in gene expression, with an 80% reduction at 0.25 mg/mL and a 90% reduction at 0.50 mg/mL, both observed at 6 h post-injection. Additionally, the mRNA expression of *HbREF* in samples silenced with *HbSRPP* dsRNA showed a slight decrease, but it was not statistically significant. Conversely, the expression of *HbSRPP* in plants silenced for *HbREF* remained unchanged. These results indicate that the dsRNA did not have off-target effects on the other gene.

### 2.4. Gene Expression Profiles in Rubber Synthesis

**Figure 4:**
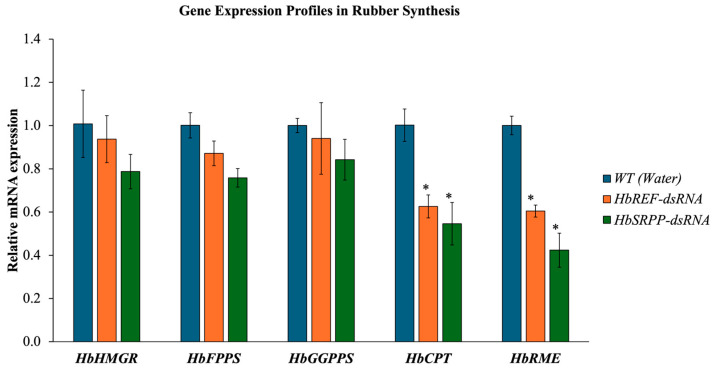
Impact of *HbREF* and *HbSRPP* gene silencing on rubber biosynthesis pathway gene expression. Quantitative RT-PCR analysis of rubber synthesis-related genes following RNAi-mediated silencing of *HbREF* and *HbSRPP* in *H. brasiliensis*. Relative mRNA expression levels of upstream genes (*HbHMGR*, *HbFPPS*, *HbGGPPS*) and downstream genes (*HbCPT*, *HbRME*) were assessed post-dsRNA treatment. Gene expression was normalized to housekeeping genes and presented relative to controls. Data represent mean ± standard error from three independent biological replicates. Statistical significance determined by one-way ANOVA (* *p* < 0.05).

The expression of genes involved in rubber synthesis was analyzed following the silencing of *HbREF* and *HbSRPP*. The results indicated that the relative expression levels of *HbHMGR*, *HbFPPS*, and *HbGGPPS* remained largely unchanged (Figure 4). In contrast, the expression of *HbCPT* and *HbRME* showed a significant reduction, with *HbCPT* decreasing by 37.43% and 45.41% when *HbREF* and *HbSRPP* were inhibited, respectively. Similarly, *HbRME* expression decreased by 39.53% to 57.62%. These findings suggest that silencing *HbREF* and *HbSRPP* also leads to a reduction in *HbCPT* and *HbRME* expression. 

### 2.5. Dry Rubber Content After Gene Silencing

**Figure 5:**
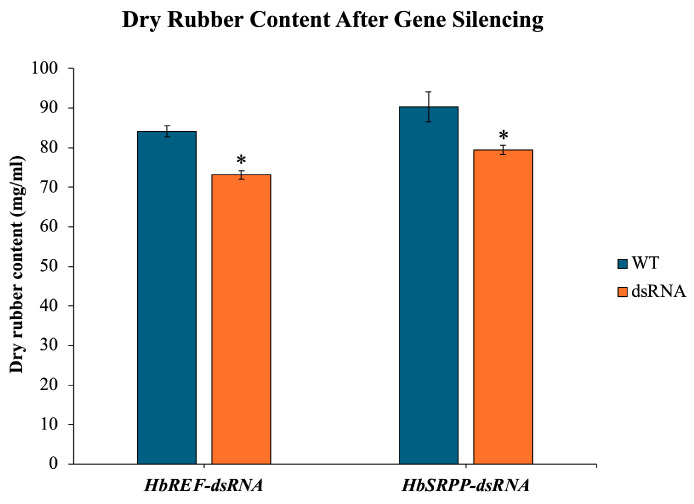
Impact of *HbREF* and *HbSRPP* gene silencing on DRC in RRIM600 saplings. Quantitative analysis of DRC following RNAi-mediated silencing of *HbREF* and *HbSRPP* genes in *H. brasiliensis* cv. RRIM600. Latex samples were collected and processed for DRC determination. Data represent mean ± standard error from six independent saplings per treatment group. Statistical significance was determined by one-way ANOVA with Tukey’s post hoc test (* *p* < 0.05).

The DRC in RRIM600 rubber tree saplings was assessed following RNAi treatment. The DRC showed a decrease of 13.07% in the *HbREF* RNAi group and 11.99% in the *HbSRPP* RNAi group compared to the control, indicating that the silencing of both *HbREF* and *HbSRPP* genes significantly reduced the DRC (Figure 5).

### 2.6. Allergenic Protein Content After Gene Silencing

**Figure 6:**
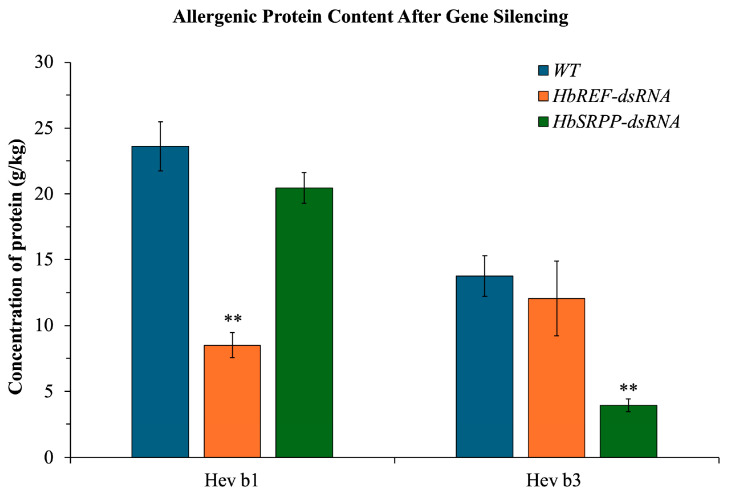
Reduction in major latex allergens following targeted gene silencing in *H. brasiliensis*. Quantitative analysis of allergenic proteins Hev b1 and Hev b3 in latex samples following RNAi-mediated silencing of *HbREF* and *HbSRPP* genes. Protein concentrations were determined by enzyme-linked immunosorbent assay (ELISA) using allergen-specific antibodies. Data represent mean ± standard error from six biological replicates. Statistical significance determined by two-way ANOVA with Tukey’s multiple comparison test (** *p* < 0.01).

After silencing the *HbREF* and *HbSRPP* genes, latex samples were collected for protein extraction. It was found that in the samples where the *HbREF* gene was silenced, the amount of Hev b1 protein decreased by nearly 64.04%, and Hev b3 protein levels also decreased by 12.51%. In contrast, in the samples where the *HbSRPP* gene was silenced, the amount of Hev b3 protein decreased by 71.54%, and Hev b1 protein levels decreased by 13.48% (Figure 6).

## 3. Discussion

The use of RNA interference (RNAi) in plants has emerged as a powerful tool for silencing specific genes to study their function or to develop traits of interest. In this study, the silencing of *HbREF* and *HbSRPP* genes in para rubber trees via dsRNA delivery methods provided valuable insights into their roles in rubber biosynthesis and potential applications for improving rubber tree traits.

In the present, there are significant implications for the application of RNAi in agricultural biotechnology, particularly in the context of improving crop traits through gene silencing. The stability of dsRNA under various environmental conditions is a critical factor that influences the effectiveness of RNAi, especially when dsRNA is applied through methods such as foliar spray or root drenching, which are common in field conditions. The stability of dsRNA is crucial because it determines how long the dsRNA can remain active in the environment before degrading, which directly impacts its efficacy in gene silencing. The study demonstrated that dsRNA is highly stable at temperatures between 25 °C and 37 °C for up to 6 h, which is encouraging for its use in various climates and conditions. This stability is particularly important because it suggests that dsRNA can maintain its integrity and functionality during the time it takes to be absorbed by plant tissues and trigger the RNAi mechanism. However, the study also found that at higher temperatures (50–60 °C), dsRNA degrades rapidly, with more than 45% degradation occurring within 6 h. This finding is significant because it highlights a potential limitation in using dsRNA-based treatments in very hot climates or during periods of extreme heat, where the efficacy of the treatment could be compromised. The degradation of dsRNA at high temperatures could limit the window of time during which it can effectively silence target genes, necessitating the consideration of timing and environmental conditions when applying dsRNA in the field. The stability of dsRNA under continuous UV irradiation for 6 h further underscores its resilience in outdoor conditions, where exposure to sunlight is inevitable. This finding is particularly important because it suggests that dsRNA can be used in open field conditions without significant degradation from sunlight, making it a viable option for foliar sprays or other surface applications. Additionally, the fact that dsRNA stored at indoors remained stable for 72 h, and did not degrade in outdoor conditions (regardless of sunlight exposure) within the same time frame, further supports its practical use in agricultural settings. These findings align with previous studies that have reported the stability of dsRNA in various conditions, emphasizing its potential for use in gene silencing applications. For example, studies by Li et al. [15], Xue et al. [16], Yang et al. [17], San Miguel and Scott [18], and Venu et al. [19] have shown that dsRNA can be stable under a range of environmental conditions, which is essential for its use in pest control and crop improvement strategies [20,21]. The ability to maintain dsRNA stability across different environments ensures that the RNAi effect can be reliably achieved in field conditions, enhancing the practical applicability of this technology. However, in this study, dsRNA stability was assessed mainly by A260 absorbance, a common preliminary method for RNA integrity [22,23]. While useful for monitoring overall degradation trends, it does not confirm molecular integrity or sequence-specific stability. In vivo, dsRNA persistence is affected by plant uptake, enzymatic degradation, soil microbiota, and environmental factors. Therefore, these in vitro results serve as preliminary guidance for application timing and delivery methods. Future studies should monitor dsRNA stability in planta and explore formulation strategies (e.g., nanoparticles or protective additives) to improve longevity and efficacy under field conditions.

In real agricultural settings, dsRNA stability is influenced not only by temperature and UV exposure but also by complex interactions with soil microbiota, plant exudates, and environmental pH. Several studies have demonstrated that dsRNA applied to soil or leaf surfaces can undergo rapid enzymatic degradation—for example, *DvSnf7* dsRNA in soil was almost completely degraded within approximately two days [24]. Conversely, formulations with protective carriers such as nanomaterials (e.g., cationic polymers, liposomes) have been shown to enhance dsRNA longevity and uptake [17,25]. Our findings that dsRNA remained stable for several hours under moderate temperatures and UV exposure provide encouraging evidence for its short-term use in open-field applications. However, long-term stability and bioavailability in heterogeneous environments remain critical challenges. Therefore, further studies integrating field trials and protective formulation strategies (e.g., nanoformulations) are needed to translate laboratory observations into robust field-level applications.

The comparison of different dsRNA delivery methods including direct injection, foliar spray, and root drenching reveals important insights into the efficiency of gene silencing in para rubber trees. The fact that all three methods were capable of delivering dsRNA into cells and achieving gene knockdown highlights the versatility of RNAi as a tool for gene regulation in plants. However, the varying degrees of effectiveness observed with each method are crucial for optimizing RNAi applications in different contexts. Direct injection emerged as the most effective method, achieving a 90% reduction in *HbREF* and *HbSRPP* expression within 12 h post-injection. This high level of efficiency is likely due to the direct introduction of dsRNA into the vascular system of plant, ensuring that a substantial amount of dsRNA reaches the target cells. The rapid onset of gene silencing observed with this method underscores its potential for applications where quick results are needed, such as in research settings or in situations where immediate intervention is required. In contrast, the foliar spray method resulted in lower gene silencing efficiency, with *HbREF* and *HbSRPP* expression reduced by 69.5% and 45.6, respectively. The leaf surface may be less efficient or slower than direct injection. This finding is consistent with previous studies that have reported challenges in achieving efficient dsRNA uptake through foliar applications, particularly due to barriers such as the cuticle and cell walls [18,26,27]. Despite these limitations, foliar spraying remains a non-invasive and scalable method that could be optimized through the use of surfactants or other adjuvants to enhance dsRNA penetration and uptake. Root drenching was also effective, reducing *HbREF* and *HbSRPP* expression by 83.7% and 54.6%, respectively. This method benefits from the natural uptake of dsRNA through the root system, which is then translocated to the leaves where gene silencing occurs. Root drenching could be particularly useful in scenarios where systemic gene silencing is desired, as the dsRNA can potentially reach various parts of the plant. However, the efficiency of this method may depend on factors such as root architecture, soil composition, and the ability of dsRNA to remain stable and mobile within the soil matrix. These findings are consistent with other studies that have compared dsRNA delivery methods in plants. For instance, Joga et al. [28] reported that direct injection was more effective than topical applications in achieving gene silencing in cotton plants. Similarly, Majidiani et al. [29] found that root absorption of dsRNA led to a 47–69% reduction in gene expression and a mortality rate of 67.1–80.5% in treated specimens. Moreover, highlighted that root delivery methods are feasible for field applications, allowing for effective pest management strategies [30].

The expression of *HbREF* and *HbSRPP* genes in para rubber tree leaves following RNAi induction offer significant insights into the molecular mechanisms underlying rubber biosynthesis. The efficient silencing of *HbREF* and *HbSRPP* using dsRNA demonstrates the potential of RNA interference (RNAi) technology as a powerful tool for gene regulation in plants, particularly in economically important crops like rubber trees. The results showed that dsRNA targeting *HbREF* achieved a dramatic reduction in gene expression, with a 98–99% decrease observed as early as 3 h post-injection. This rapid and substantial knockdown of *HbREF* highlights the effectiveness of RNAi in targeting specific genes involved in rubber biosynthesis. The ability to achieve such a high level of gene silencing within a short timeframe is crucial for applications where timely intervention is necessary, such as in the study of gene function or the development of genetically modified crops with enhanced traits. For *HbSRPP*, the study observed a dose-dependent response to dsRNA, with the highest concentration (0.065 mg/mL) reducing gene expression by up to 70%, and even greater reductions observed with increased dsRNA concentrations (80% at 0.25 mg/mL and 90% at 0.50 mg/mL). These findings suggest that *HbSRPP* expression can be effectively controlled through RNAi, allowing researchers to modulate rubber particle stability and synthesis by adjusting dsRNA dosage. This dose-dependent response also underscores the flexibility of RNAi technology in achieving the desired level of gene silencing based on experimental needs. Interestingly, the study found that silencing *HbSRPP* did not significantly affect *HbREF* expression, and vice versa, indicating that the dsRNA treatments were specific to their target genes and did not produce off-target effects. This specificity is crucial for ensuring that RNAi technology can be used to manipulate gene expression without unintended consequences on non-target genes, which is a major concern in genetic engineering and crop improvement. It should also be noted that the silencing efficiency observed from direct injection experiments (>90% within 12 h) appeared higher than the dose–response assays, where variability in knockdown efficiency was detected. This discrepancy could be attributed to differences in dsRNA uptake and distribution between experimental setups. In direct injection, dsRNA is introduced immediately into the vascular tissues, enabling rapid and efficient delivery to the target cells. By contrast, the dose–response assays required a more gradual cellular uptake, during which degradation or dilution of dsRNA may have occurred, leading to variable outcomes. Moreover, experimental variation in tissue penetration and intracellular trafficking may also contribute to the observed inconsistency. These factors highlight the importance of considering delivery routes and cellular dynamics when interpreting RNAi efficiency, and future work comparing in vivo dsRNA stability and tissue distribution will be essential to reconcile these differences.

In addition to silencing *HbREF* and *HbSRPP*, the study examined the expression of other genes involved in rubber synthesis, such as *HbHMGR*, *HbFPPS*, *HbGGPPS*, *HbCPT*, and *HbRME*. The findings that *HbHMGR*, *HbFPPS*, and *HbGGPPS* expression levels remained largely unchanged after *HbREF* and *HbSRPP* silencing suggest that these upstream genes in the rubber biosynthesis pathway are not directly dependent on *HbREF* and *HbSRPP*. This result is important because it indicates that the initial stages of rubber biosynthesis, specifically the production of key precursors like isoprenoids, are not disrupted by the silencing of *HbREF* and *HbSRPP*. This stability in precursor production may allow the rubber tree to maintain basic metabolic functions even when the downstream components involved in rubber particle formation are disrupted. However, the significant reduction in *HbCPT* and *HbRME* expression following *HbREF* and *HbSRPP* silencing is particularly noteworthy. HbCPT plays a crucial role in catalyzing the polymerization of isoprene units to form polyisoprene chains, which are the primary components of natural rubber. The observed decrease in *HbCPT* expression (37.43% and 45.41% for *HbREF* and *HbSRPP* silencing, respectively) suggests that *HbREF* and *HbSRPP* are closely linked to the regulation of *HbCPT*. This reduction likely impairs the rubber biosynthesis process, as *HbCPT* is essential for the elongation of rubber molecules. Similarly, the reduction in *HbRME* (Rubber Membrane Elongation Protein) expression by 39.53% to 57.62% following *HbREF* and *HbSRPP* silencing suggests that these proteins are also involved in maintaining the structural integrity of rubber particles. HbRME is thought to play a role in the elongation and stability of the membrane surrounding rubber particles, which is crucial for the proper synthesis and accumulation of rubber. The reduction in *HbRME* expression indicates that disrupting *HbREF* and *HbSRPP* not only affects rubber biosynthesis at the molecular level but also impacts the physical properties of the rubber particles themselves.

This study align with previous research that has highlighted the importance of REF and SRPP in rubber biosynthesis. Dennis and Light [31] first identified REF as a key protein involved in rubber particle elongation, and subsequent studies have confirmed its role in stabilizing and promoting the growth of rubber particles [32,33]. The dramatic reduction in *HbREF* expression observed in this study further supports the critical function of REF in rubber biosynthesis. Similarly, the role of SRPP in stabilizing small rubber particles has been well-documented [34,35,36]. The dose-dependent silencing of *HbSRPP* in this study provides new insights into how varying levels of SRPP influence rubber particle stability and synthesis. The ability to fine-tune *SRPP* expression through RNAi could have important implications for optimizing rubber yield and quality. The reduction in *HbCPT* expression observed in this study is consistent with findings from Priya et al. [37], who reported that the downregulation of genes involved in polyisoprene chain elongation leads to decreased rubber production. This study’s results extend these findings by showing that *HbCPT* expression is closely tied to REF and SRPP levels, further elucidating the complex network of interactions that govern rubber biosynthesis. Overall, the study’s findings contribute to a growing body of literature on the molecular mechanisms of rubber biosynthesis and highlight the potential of RNAi as a tool for regulating gene expression in rubber trees. By demonstrating the specific and dose-dependent effects of dsRNA on *HbREF* and *HbSRPP* expression, the study provides a foundation for future research aimed at improving rubber production through targeted genetic interventions.

The RNAi-mediated silencing of *HbREF* and *HbSRPP* in *H. brasiliensis* saplings provides clear evidence that both genes play crucial roles in rubber biosynthesis and allergenic protein production. The observed reductions in DRC (13.07% for *HbREF*, 11.99% for *HbSRPP*) indicate that both proteins contribute significantly and almost equally to rubber yield, consistent with previous findings that downregulation of similar proteins compromises rubber particle stability and biosynthesis efficiency [13,38]. At the same time, silencing differentially affected allergenic proteins: *HbREF* knockdown reduced Hev b1 by 64.04% and Hev b3 by 12.51%, while *HbSRPP* knockdown led to a 71.54% reduction in Hev b3 and a 13.48% decrease in Hev b1, reinforcing their involvement in allergenic protein synthesis [39,40].

Importantly, the magnitude of allergenic protein reduction was far greater than the relatively modest decline in rubber yield. This suggests a favorable trade-off for specialized applications. A 12–13% reduction in yield may be commercially tolerable if it results in latex with substantially lower allergenic potential, particularly for medical products where latex allergy poses significant risks. The results underscore the balance between maintaining rubber productivity and minimizing allergenic proteins, highlighting RNAi as a promising strategy for producing hypoallergenic latex while still preserving viable levels of rubber production.

This study represents the first demonstration of targeted silencing of *HbREF* and *HbSRPP* in para rubber trees using exogenous dsRNA delivery. The dual outcome—simultaneous reduction in allergenic proteins and modulation of rubber biosynthesis—highlights a novel application of RNAi in a perennial industrial crop, extending its scope beyond annual plants and insect control. These results not only advance the understanding of REF and SRPP function but also lay the foundation for developing hypoallergenic natural rubber with direct implications for medical and industrial use. Nevertheless, some limitations should be acknowledged: although no visible morphological abnormalities were observed during the experimental period, long-term effects on tree growth, yield, and latex quality remain to be determined. Future research should therefore include optimized delivery systems, long-term field evaluations, ultrastructural and physicochemical analyses of latex particles, and functional allergenicity assays. Such efforts will be essential to assess the feasibility of RNAi as a sustainable strategy for crop improvement and specialty latex production.

## 4. Materials and Methods

### 4.1. Extraction of Total RNA

Young leaves from RRIM600 para rubber trees at the C stage were harvested, weighed (0.1 to 0.3 g), and ground into a fine powder using a mortar and pestle with liquid nitrogen. Total RNA was extracted using the Plant RNA Kit (Omega Biotek, Norcross, GA, USA). The extraction process involved adding 600 µL of NTL lysis buffer to the powdered leaves, followed by vortexing and the addition of 140 µL of SP buffer. The mixture was centrifuged at 10,000× *g* for 10 min at 25 °C. The supernatant was transferred to a new tube, mixed with an equal volume of 100% isopropanol, and centrifuged again at 10,000× *g* for 2 min. The RNA pellet was air-dried for 1 min, dissolved in 100 µL of 65 °C RB buffer, and incubated at 65 °C to ensure complete dissolution.

To further purify the RNA, 250 µL of RB buffer containing 2-mercaptoethanol and 350 µL of 100% ethanol were added, and the mixture was loaded onto a HiBind^®^ RNA Mini Column (Omega Bio-tek Inc., Norcross, GA, USA). The column was washed with RNA wash buffers I and II and centrifuged at 12,000× *g*, and the RNA was eluted with 50 µL of nuclease-free water. The concentration and purity of the RNA were measured by absorbance at 260 nm (A260) and by the A260/A280 ratio, which typically ranged between 1.7 and 2.1. To remove any residual DNA, 2 µg of total RNA was treated with DNase I (Thermo Fisher Scientific Inc., Waltham, MA, USA) and incubated at 37 °C for 30 min, followed by inactivation of DNase I with 25 mM EDTA at 65 °C for 10 min.

### 4.2. Synthesis of cDNA Template

The DNA-free RNA was used to synthesize first-strand cDNA. One microgram of total RNA was mixed with 1.5 µL of 100 µM oligo (dT) primer, and the volume was adjusted to 15 µL with DEPC-treated water. The mixture was incubated at 65 °C for 5 min, then immediately cooled on ice. The reverse transcription reaction was carried out by adding 2 µL of 10× RT reaction buffer, 0.5 µL of 20 mM dNTP mix, 0.5 µL of RiboGrip RNase Inhibitor (40 U/µL), and 1 µL of FIREScript^®^ Reverse Transcriptase (200 U/µL) (Solis BioDyne, Tartu, Estonia). The reaction was incubated at 37 °C for 30 min and terminated by heating at 85 °C for 5 min. The synthesized cDNA was stored at −20 °C for use in subsequent PCR reactions.

### 4.3. dsRNA Production

To produce dsRNA, the *HbREF* or *HbSRPP* genes were amplified using specific primers designed from conserved regions in the GenBank database (Table 1). PCR was performed using GoTaq^®^ Flexi DNA Polymerase (Promega, Madison, WI, USA) in a 25 µL reaction volume with an initial denaturation at 95 °C for 5 min, followed by 34 cycles of 95 °C for 45 s, 60 °C for 45 s, and 72 °C for 1 min, with a final extension at 72 °C for 5 min. PCR products were resolved on a 1% agarose gel, and the desired DNA fragments were purified using the BioFACT^TM^ Gel & PCR Purification System (Daejeon, Republic of Korea).

The purified gene fragments were ligated into the pGEM^®^-T Easy vector and transformed into *E. coli* DH5alpha cells. Colonies containing recombinant plasmids were selected using blue-white screening, and plasmids were extracted and validated by sequencing. For dsRNA synthesis, the validated plasmids were introduced into *E. coli* HT115 (DE3). dsRNA production was induced with 1 mM IPTG for 4 h at 37 °C with shaking at 200 rpm, and the dsRNA was extracted using TRIzol reagent (Thermo Fisher Scientific Inc., Waltham, MA, USA). The RNA pellet was resuspended in 500 µL of 0.3 M NaCl prepared in DEPC-treated water. The RNA was then incubated at 65 °C for 30 min and allowed to gradually cool to room temperature to facilitate strand annealing into double-stranded RNA (dsRNA). Subsequently, 5 µL of 100 µg/mL RNase A (Thermo Fisher Scientific Inc., Waltham, MA, USA) (diluted in 0.3 M NaCl) and 5 µL of 500 µg/mL DNase I were added, and the mixture was incubated at 37 °C for 30 min to remove residual single-stranded RNA and DNA. The integrity of the resulting dsRNA was analyzed by 1% agarose gel electrophoresis. The dsRNA concentration was determined by measuring absorbance at 260 nm (A260), and purity was evaluated from the A260/A280 ratio, which ranged between 1.7 and 2.1.

In future studies, in vitro transcription could be employed as an alternative RNAi resource to obtain highly pure and reproducible dsRNA, which may further improve experimental consistency.

### 4.4. Degradation Test

The stability of dsRNA targeting *HbREF* and *HbSRPP* was assessed under various environmental conditions to provide preliminary guidance for application strategies. A total of 50 µL samples of 10 ng/µL dsRNA were keeped into sterilized PCR tubes which sealed with Parafilm^®^ M (Amcor PLC, Zürich, Switzerland), and the tubes were tested to various treatments, including storage at 4 °C, placement in a 25, 37, 40, 50 and 60 °C water bath. A total of 50 µL samples of 15 ng/µL dsRNA were exposure to direct outdoor sunlight, and shade for 10, 90, 180, and 360 min. Additionally, 50 µL samples of 15 ng/µL dsRNA were placed in plates without sealing and exposed to UV lamp, 365 nm, 15 W, at distance of 20 cm for 10, 90, 180, and 360 min. After each treatment, dsRNA concentrations were quantified using absorbance at 260 nm (A260) with a SPECTROstar Nano microplate reader equipped with LVis Plate technology (BMG Labtech, Ortenberg, Germany), and the degradation rate was calculated as:

The degradation rate = [(initial concentration − final concentration)/initial concentration] × 100(1)

Each treatment included three independent biological replicates. While this method allows quantitative assessment of dsRNA integrity, we acknowledge that absorbance measurements cannot distinguish between intact dsRNA and partial degradation fragments, nor do they capture potential sequence-specific effects. These in vitro assays therefore provide preliminary stability trends under controlled conditions rather than precise predictions of in vivo dsRNA performance.

### 4.5. Comparison of Delivery Techniques

The efficiency of different dsRNA delivery methods (foliar spray, root drenching, and injection) in silencing target genes in para rubber tree leaves was compared. Each method was applied using dsRNA targeting specific genes (*HbREF* and *HbSRPP*) at a concentration of 0.500 mg/mL. For the foliar spray, a fine mist sprayer was used to apply the dsRNA solution mixed with 0.1% (*v*/*v*) Tween 20 uniformly to the leaves. In the root drenching method, the dsRNA solution was poured evenly around the base of the plants to ensure uptake by the roots. In the injection method, the dsRNA was directly injected into the leaf veins using a fine needle.

Leaf samples were collected from each group at 12 h post-application to evaluate dsRNA uptake and the silencing efficiency of the target genes. Total RNA was extracted from the collected leaves, and cDNA was synthesized through reverse transcription. The synthesized cDNA was then used as a template for reverse transcription quantitative PCR (RT-qPCR). The relative expression levels of the target genes were determined using RT-qPCR, with *β-actin* as the internal control for normalization. Each measurement included three biological replicates, with three technical replicates per sample. The qPCR reaction mixture included 1× HOT FIREPol^®^ EvaGreen^®^ qPCR Mix Plus (ROX) (Solis BioDyne, Tartu, Estonia), 0.5 µM of each forward and reverse primer (Table 1), and the first-strand cDNA. Real-time PCR performed on StepOnePlus™ (Applied Biosystems, Waltham, MA, USA) under the following conditions: initial denaturation at 95 °C for 12 min, followed by 40 cycles of 95 °C for 15 s, 60 °C for 20 s, and 72 °C for 20 s. A melting curve analysis was conducted after the amplification cycles, ranging from 60 °C to 95 °C, with a gradual temperature increase to verify the specificity of the amplified product. Each treatment included three independent biological replicates (three independent saplings per treatment group), each with three technical replicates per sample.

### 4.6. Gene Silencing of HbREF and HbSRPP

Rubber tree were divided into seven groups: Groups 1–3 were injected with *HbREF* dsRNA at concentrations of 0.035, 0.050, and 0.065 mg/mL, respectively; Groups 4–6 received *HbSRPP* dsRNA at the same concentrations; Group 7 served as the control and was injected with distilled water. Injections were administered directly into the leaf veins, with 100 µL of solution per leaf.

To assess the initial response to gene silencing, leaf samples were collected at 3, 6, and 12 h post-injection for analysis. Total RNA was extracted from the samples, reverse transcribed into cDNA, and real-time PCR was conducted using gene-specific primers for *HbREF* and *HbSRPP*, with *β-actin* serving as the internal control. Additionally, to evaluate potential off-target effects in the RNAi experiments targeting *HbREF* or *HbSRPP*, the mRNA expression of the other gene (*HbSRPP* or *HbREF*) was also examined. The cycle threshold (C_T_) values obtained were used to calculate the relative expression levels of the target genes. Each time point included three independent biological replicates (three independent saplings per treatment group), each with three technical replicates per sample.

### 4.7. Gene Expression Analysis

To investigate the impact of gene silencing of *HbREF* and *HbSRPP*, several key genes involved in rubber particle synthesis in rubber trees were selected for analysis of their expression profiles post-silencing. The selected genes included *HbCPT* (Cis-prenyltransferase), HbHMGR (3-Hydroxy-3-Methylglutaryl-CoA Reductase), *HbFPPS* (Farnesyl Pyrophosphate Synthase), *HbGGPPS* (Geranylgeranyl Pyrophosphate Synthase), and *HbRME*. Total RNA was extracted from the samples, converted into cDNA via reverse transcription, and the expression levels of these genes were analyzed using RT-qPCR with gene-specific primers (Table 1). *β-actin* was used as the internal control for normalization. All experiments were performed in three independent biological replicates (three independent saplings per treatment group), each with three technical replicates per sample.

### 4.8. Determination of Dry Rubber Content

To assess the impact of RNAi on latex production, the DRC was measured. Latex samples were collected at 24 h post-injection from six independent saplings per treatment group. Approximately, 20 µL of latex was mixed with 100 µL of rubber extraction buffer (REB) containing 5 mM DTT. The mixture was centrifuged at 12,000× *g* for 20 min at 4 °C to coagulate the rubber particles. The coagulate was washed with methanol, dried for 24 h, and weighed to determine the DRC. Data are presented as mean ± standard error (SE) from six independent saplings per treatment group.

### 4.9. Determination of Allergenic Protein Content

To confirm the efficacy of RNAi, latex samples were collected at 24 h post-injection from six independent saplings per treatment group and mixed with PBS at a ratio of 1:5. The protein extraction was carried out at room temperature for 2 h in a shaking incubator. Following extraction, the samples were centrifuged at 2000× *g* for 15 min, and the supernatant was collected for further analysis. The concentrations of Hev b1 and Hev b3 proteins were quantified using an ELISA with the FIT kit (Icosagen AS, San Francisco, CA, USA). In brief, 100 µL of assay buffer was dispensed into each well of the microplate pre-coated with antibodies specific for Hev b1 or Hev b3. Then, 25 µL of the protein extract from latex was added to the wells and incubated for 60 min at room temperature with shaking at 200 rpm. After incubation, the wells were washed four times with the washing solution. Next, enzyme conjugate was added to each well and incubated for 30 min at room temperature with shaking at 200 rpm. The wells were again washed four times with the washing solution. HRP substrate was then added to each well, and the plate was incubated for 15 min at room temperature with shaking at 200 rpm. After incubation, the reaction was stopped by adding the stop solution, and the plate was shaken gently for 1–2 min. Absorbance was measured at 414 nm, and the concentrations of Hev b1 and Hev b3 were calculated using standard curves prepared with Hev b1 and Hev b3 standards in the range of 10–1000 µg/L. Data represent mean ± SE from six biological replicates (six independent latex samples per treatment group), each measured in three technical replicates.

## 5. Conclusions

This study demonstrates that the RNAi-mediated silencing of *HbREF* and *HbSRPP* genes in *H. brasiliensis* effectively reduces major latex allergens (Hev b1 and Hev b3) while preserving rubber production at acceptable levels. Notably, the approach achieves up to 71% allergen reduction with only 13% decrease in DRC, indicating a favorable therapeutic index and strong commercial potential for developing hypoallergenic latex products. This research further provides novel insights into dsRNA stability under environmental conditions, showing that both *HbREF*- and *HbSRPP*-specific dsRNA maintain functional integrity under moderate temperature, sunlight, and UV exposure. Additionally, the comparative evaluation of delivery methods (direct stem injection, foliar spray, and root drenching) reveals flexible and efficient strategies for RNAi application in perennial crops. Collectively, these findings underscore the innovative aspect of integrating gene-specific RNAi with environmentally resilient delivery systems, establishing a robust biotechnological framework for precision modulation of latex allergenicity. This study offers a significant advance in hypoallergenic latex production, paving the way for safer latex products and providing a model for RNAi-based trait improvement in other perennial crop species.

## Figures and Tables

**Table 1:** Oligonucleotide primers used in this study.

Primers	Sequence (5′-3′)	Usage
*HbREF*-F1	ATGGCTGAAGACGAAGACAAC	dsRNA production
*HbREF*-R1	CCCTCAATGATATCGACACCA	dsRNA production
*HbSRPP*-F1	AGACAAAACGGTGGATGTATCG	dsRNA production
*HbSRPP*-R1	GCTTTTGGTTCAAGATTAGCGT	dsRNA production
*HbREF*-F2	CTGGTCCATTCAAGCCTGGCG	RT-qPCR
*HbREF*-R2	GGAGCGAACGACCTAACACCGAACT	RT-qPCR
*HbSRPP*-F2	TGGCTGAAGAGGTGGAAGAGAGG	RT-qPCR
*HbSRPP*-R2	AGGAGTAACCACGGTCTTCACCAC	RT-qPCR
*HbHMGR*-F	GGAAGTGGGTCCTGATTTCC	RT-qPCR
*HbHMGR*-R	CAAGGCCATCCTCTGTCGTA	RT-qPCR
*HbFPPS*-F	AGCAGTACCGGAGGAGGAAG	RT-qPCR
*HbFPPS*-R	CTTGGCAGCAGTCTGGAATA	RT-qPCR
*HbGGPPS*-F	CTTCGTGGAGCTGGTGTTCT	RT-qPCR
*HbGGPPS*-R	AGGACTTGTTGGCATTGAGG	RT-qPCR
*HbCPT*-F	GGAGGTGGTCATAAGGCTGGATTT	RT-qPCR
*HbCPT*-R	TGGAATTGTTGGCAGTAGCCCT	RT-qPCR
*HbRME*-F	GCTCTGACAGGAGAGGAAAC	RT-qPCR
*HbRME*-R	GACGATGGTGATGAGGAGGA	RT-qPCR
*β-actin*-F	GCACACGATCCAACACCACCAA	RT-qPCR
*β-actin*-R	TAGACTGATGCCTGGGGCCTGA	RT-qPCR

## Data Availability

The original contributions presented in this study are included in the article. Further inquiries can be directed to the corresponding author.

## Abbreviations

The following abbreviations are used in this manuscript:

DRC	Dry rubber content
*DvSnf7*	Suppressor of nedd4-1–binding factor 7 gene from *Diabrotica virgifera*
*HbCPT*	Cis-prenyltransferase gene from *H. brasiliensis*
*HbFPPS*	Farnesyl Pyrophosphate Synthase gene from *H. brasiliensis*
*HbGGPPS*	Geranylgeranyl Pyrophosphate Synthase gene from *H. brasiliensis*
*HbHMGR*	3-Hydroxy-3-Methylglutaryl-CoA Reductase gene from *H. brasiliensis*
*HbRME*	Rubber Membrane Elongation Protein gene from *H. brasiliensis*
*HbREF*	Rubber elongation factor gene from *H. brasiliensis*
*HbSRPP*	Small rubber particle protein gene from *H. brasiliensis*
Hev b1	Rubber elongation factor (REF), a major protein allergen from natural rubber latex of *H*. *brasiliensis*
Hev b3	Small rubber particle protein (SRPP), a major protein allergen from natural rubber latex of *H. brasiliensis*
HRP	Horseradish peroxidase
REF	Rubber elongation factor
SRPP	Small rubber particle protein
UV	Ultraviolet
